# Tissue-specific responses of antioxidant pathways to poor hygiene conditions in growing pigs divergently selected for feed efficiency

**DOI:** 10.1186/s12917-019-2107-2

**Published:** 2019-10-16

**Authors:** K. Sierżant, M-H. Perruchot, E. Merlot, N. Le Floc’h, F. Gondret

**Affiliations:** 1 Faculty of Biology and Animal Science, Department of Animal Nutrition and Feed Science, Wroclaw University of Environmental and Life Sciences, 51630 Wroclaw, Poland; 2 PEGASE, INRA, AGROCAMPUS OUEST, 35590 Saint-Gilles, France

**Keywords:** Adipose tissue, Antioxidant enzymes, Feed efficiency, Hygienic challenge, Oxidative stress

## Abstract

**Background:**

Poor hygiene of housing induces a systemic inflammatory response. Because inflammation and oxidative stress are processes that can sustain each other, the ways pigs are able to activate their antioxidant defenses are critical for production performance and health during periods when the immune system is solicited. Selection for production performance can also influence reactive oxygen species (ROS) production and expression levels of genes involved in cellular response to oxidative stress in different tissues. To establish the extent by which poor hygiene and selection for feed efficiency affected redox status, pigs divergently selected for residual feed intake (RFI) were housed in poor or good hygiene during 6 weeks. At the end, blood was collected in all pigs, and half of them were killed for tissue sampling. The remaining pigs were reared in good hygiene conditions during a recovery period of 7–8 weeks.

**Results:**

At week 6, poor hygiene was associated with a lower total antioxidant capacity assessed by plasma ferric reducing ability in all pigs, and with greater plasma levels of hydrogen peroxides in the high RFI pigs (less efficient). Adipose tissue of high RFI pigs exhibited higher activities of catalase and glutathione reductase, and greater thiobarbituric acid reactive substances (TBARS) concentrations when compared with the low RFI pigs (more efficient). Poor hygiene conditions activated the antioxidant enzymes activities (glutathione reductase, superoxide dismutase and catalase) in adipose tissue of both lines, but led to higher ROS production by mature adipocytes isolated from the high RFI pigs only. In liver and muscle, there were only minor changes in antioxidant molecules due to genetics and hygiene conditions. After the resilience period, adipose tissue of pigs previously challenged by poor hygiene maintained higher antioxidant enzyme activities, and for the high RFI line, displayed higher TBARS concentrations.

**Conclusions:**

Pigs selected for improved feed efficiency showed a lower susceptibility to oxidative stress induced by poor hygiene conditions. This could led to a lower inflammatory response and less impaired growth when these pigs are facing sanitary challenges during the production period.

## Background

Poor hygiene and the non-respect of biosecurity rules in commercial farms favor the occurrence of diseases and subclinical diseases. The housing environment through the continual exposition to dust, ammonia, microbial and antigens pressure is thus a determinant of production performance and health of pigs [1, 2]. Poor hygiene of housing impairs nutrient digestibility, feed intake, growth rate, and *in fine*, feed efficiency [3, 4]. It also induces a systemic inflammatory response, so that elevated concentration of haptoglobin, an acute phase protein found in plasma during inflammation and infection, and higher numbers of lymphocytes and granulocytes have been observed in blood formula of pigs housed in poor hygiene conditions [4, 5]. Inflammation and oxidative stress are correlated physiological processes. Indeed, superoxide radicals are formed by the NADPH oxidase from activated immune cells during inflammatory reactions, while oxidants are activators of the NF-κB pathway triggering inflammation [6]. Oxidative stress arises when the generation of reactive oxygen species (ROS) overwhelm the endogenous antioxidant defenses. These defenses rely on molecules of high (enzymes) and low (glutathione and vitamins) molecular weights, which can reduce the formation and/or scavenge oxidants and prevent oxidative stress.

Importantly, genetic selection for production performance traits may also affect mitochondrial functionality, ROS production, and the expression profiles of genes involved in the cellular response to oxidative stress. Among production traits, feed efficiency has received regain interest because 60–70% of total production costs in farm animals is based on feed. Higher levels of ROS production and oxidized mitochondrial proteins have been found in muscle of low feed efficient chickens [7], and the studies also suggest the existence of tissues-specific regulatory mechanisms on mitochondria protein expressions in these animals. In healthy and resting pigs, ROS production in mitochondria was higher in *semitendinosus* muscle of the less efficient pigs selected for high residual feed intake (RFI) when compared with more efficient pigs selected for low RFI [8]. However, hydrogen peroxides production was similar in the *longissimus* muscle and liver of both lines [8]. Genes coding for antioxidant enzymes also exhibited differential expression levels in healthy pigs from the high or low RFI lines, and differences between lines were tissue-specific [9]. When housed in degraded hygiene conditions, growth performance and health were more affected in the high RFI pigs than in the low RFI pigs [5]. We hypothesized that these differences to cope with the inflammation generated by poor environmental hygiene may be due to line-associated differences in the ability to cope with oxidative stress. Therefore, the objectives of this study were to investigate oxidative production and antioxidant capacities of genetically divergent RFI pigs considered in basal and immune-stimulated conditions. The way different tissues participated to mitigate oxidative stress when those pigs were facing poor hygiene conditions, was also considered.

## Results

Pigs from two lines divergently selected for low (LRFI) or high (HRFI) residual feed intake (RFI) were housed either in good or poor hygiene conditions during the first 6 weeks (W) after their transfer in growing pens (period 1, challenge). Half of these pigs were killed at W6, while another half were placed in good hygiene conditions (period 2, resilience) until slaughter at W13 to W14. At each slaughter stage, carcass, digestive tract, lung, and snout were carefully inspected. Any pathological lesions such as pericarditis and pleurisy were recorded together with the prevalence of pneumonia. Details on the effects of the environmental hygiene degradation on systemic inflammation, growth performance and the prevalence and severity of pulmonary lesions at slaughter can be found in our associate publication [5]. In brief, poor hygiene conditions were associated with a higher prevalence of pneumonia and lung lesions at the end of period 1 in all pigs, but health of the most efficient pigs (LRFI) was less impaired.

### Pig performance and body composition

The HRFI pigs grew slower during period 1 (W0 to W6) and were lighter than the LRFI pigs at the end of this period (Table 1). Although initial body weight (BW) influenced (*P* < 0.01 to *P* < 0.07) growth performance, covariate analysis attested that the differences between groups were not solely due to difference in initial BW but were also inherent (*P* < 0.001) to RFI genetic lines. Importantly, pigs housed in poor hygiene conditions exhibited a lower BW at the end of period 1 when compared with pigs housed in good conditions. Daily growth rate from W0 to W6 was reduced due to hygiene degradation in the HRFI pigs, with no significant difference in the LRFI pigs (Line x Hygiene, *P* = 0.08). In period 2 (W6 to W12), there was no more difference in daily growth rate between the two lines, but at the end, the HRFI pigs still tended to be lighter (*P* ≤ 0.10) than the LRFI pigs. In addition, pigs housed in poor hygiene conditions during period 1 were lighter (*P* < 0.01) at the end of the experiment as compared with pigs never exposed to poor hygiene.

**Table 1:** Effects of RFI line and hygiene conditions on growth performance of pigs

Line	Low RFI		High RFI			*P* values		
Hygiene	Good	Poor	Good	Poor	MSE	Line	Hygiene	LxH
Period 1 (*n* = 71)								
BW W0	27.9	28.0	24.7	25.0	3.2	**< 0.001**	0.80	0.93
BW W6	62.5	58.6	55.6	48.1	6.2	**< 0.001**	**0.006**	0.23
ADG	808c	717bc	762b	537a	115	**< 0.001**	**< 0.001**	***0.08***
Proportions of tissues								
SCAT	5.0	4.7	5.0	4.7	0.7	0.91	0.18	0.96
PRAT	0.56	0.48	0.48	0.39	0.11	**0.02**	**0.02**	0.99
Liver	2.3	2.3	2.6	2.7	0.2	**0.005**	0.56	0.71
LL	5.5bc	5.8c	5.3ab	5.1a	0.4	**0.002**	0.75	***0.07***
Period 2 (*n* = 35)								
BW W12	102.4	90.8	98.8	81.1	10.0	***0.06***	**0.002**	0.38
End BW	111.0	100.2	110.2	90.1	9.4	***0.10***	**< 0.001**	0.17
ADG	888	774	959	832	147	0.21	**0.02**	0.88
Proportions of tissues								
SCAT	6.8	6.2	6.7	5.8	1.5	0.65	0.14	0.71
PRAT	0.86	0.87	0.92	0.67	0.22	0.31	0.15	0.11
Liver	1.61	1.72	1.98	2.11	0.17	**< 0.001**	**0.04**	0.90
LL	5.7	5.6	5.5	5.3	0.6	0.20	0.39	0.53

Pigs from two lines divergently selected for low (LRFI) or high (HRFI) residual feed intake (RFI) were housed either in good or poor hygiene conditions during the first 6 weeks (W) after their transfer in growing-finishing pens (period 1, challenge). Half of these pigs were killed at week 6 (W6: *n* = 20 LRFI pigs in good conditions, n = 20 HRFI pigs in good conditions, *n* = 15 LRFI pigs in poor conditions, *n* = 16 HRFI pigs in poor conditions). Another half were placed in good hygiene conditions until slaughter at weeks 13 or 14 (period 2, recovery: *n* = 10 LRFI pigs from good conditions, *n* = 10 HRFI pigs from good conditions, *n* = 7 LRFI pigs from poor conditions, n = 8 HRFI pigs from poor conditions). Body weight (BW, kg) at week 0 (W0), 12 (W12) and just before slaughter (end BW) were individually assessed. Average daily gain (ADG, g/d) was calculated during the two periods. Tissues (LL: *longissimus lumborum* muscle; PRAT: perirenal adipose tissue, SCAT: subcutaneous adipose tissue) were weighed at slaughter, and expressed in proportion to BW. Letters (a,b,c) were added in case of interaction (LxH; *P* < 0.10) between hygiene (H) and RFI line (L), and means sharing a common letter did not differ. MSE: root mean standard error of the statistical model. Bold face highlights significant differences (*P* ≤ 0.05) between treatments, and when italicized, this denotes a trend (0.05 < *P* ≤ 0.10)

At W6 (end of period 1), the relative weights of the perirenal adipose tissue (PRAT) and *longissimus lumborum* (LL) muscle were lower (*P* < 0.05) in HRFI pigs than in LRFI pigs, but liver proportion was higher in HRFI pigs than in LRFI pigs (Table 1). The proportion of dorsal subcutaneous adipose tissue (SCAT) was similar in both lines. Absolute weights of PRAT, SCAT, liver and LL muscle can be found in Additional file 1: Table S1. Poor hygiene conditions decreased the relative weight of PRAT, without any significant changes for SCAT, LL and liver. At W13–14 (end of period 2), the proportions of adipose tissues and LL muscle were similar in the four groups of pigs. However, liver proportion was again higher in HRFI pigs than in LRFI pigs. It was also higher (*P* < 0.05) in pigs housed in poor conditions during the first period than in pigs never exposed to poor hygiene conditions.

### Plasma redox status

At W6 (end of period 1), reactive oxygen metabolites (dROM) and total antioxidant activity estimated by the ferric reducing antioxidant power (FRAP) in plasma did not differ between the LRFI pigs and HRFI pigs when housed in good hygiene conditions (Table 2). Pigs housed in poor hygiene conditions had lower FRA*P* values in plasma than pigs housed in good conditions. On the opposite, hygiene degradation increased dROM plasma levels in the HRFI pigs, but had no significant effect in the LRFI pigs (Line x Hygiene, *P* < 0.01). At W13–14 (end of period 2), dROM and FRAP levels in plasma did not differ between the four groups.

**Table 2:** Effects of RFI line and hygiene conditions on redox status in plasma of pigs

Line	Low RFI		High RFI			*P* values		
Hygiene	Good	Poor	Good	Poor	MSE	Line	Hygiene	LxH
Week 6 (*n* = 71)								
dROM	987ab	1023b	857a	1200c	233	0.68	**0.001**	**0.008**
FRAP	203	192	210	185	22	0.95	**0.002**	0.18
Week 13–14 (*n* = 35)								
dROM	1146	990	1045	1102	194	0.94	0.50	0.14
FRAP	202	232	202	207	30	0.71	0.31	0.11

Pigs from two lines divergently selected for low (LRFI) or high (HRFI) residual feed intake (RFI = were housed either in good or poor hygiene conditions during the first 6 weeks after their transfer in growing-finishing pens (period 1). Half of these pigs were killed at week 6, whereas another half were placed in good hygiene conditions until slaughter at weeks 13 or 14 (period 2). Blood was sampled from the jugular in living pigs at the end points of the two periods. Systemic reactive oxygen metabolites (dROM) were expressed as Carratelli Unit (1 CARRU = 0.08 mg H_2_O_2_/100 mL). Ferric reducing ability of plasma (FRAP), a measure of its antioxidant power, was expressed as molar Trolox equivalents per L. Letters (a,b,c) were added in case of interaction (LxH; *P* < 0.10) between hygiene (H) and RFI line (L), and means sharing a common letter did not differ. MSE: root mean standard error of the statistical model. Bold face highlights significant differences (*P* ≤ 0.05) between treatments

### Tissue lipid content and oxidized lipid products

**Fig. 1:**
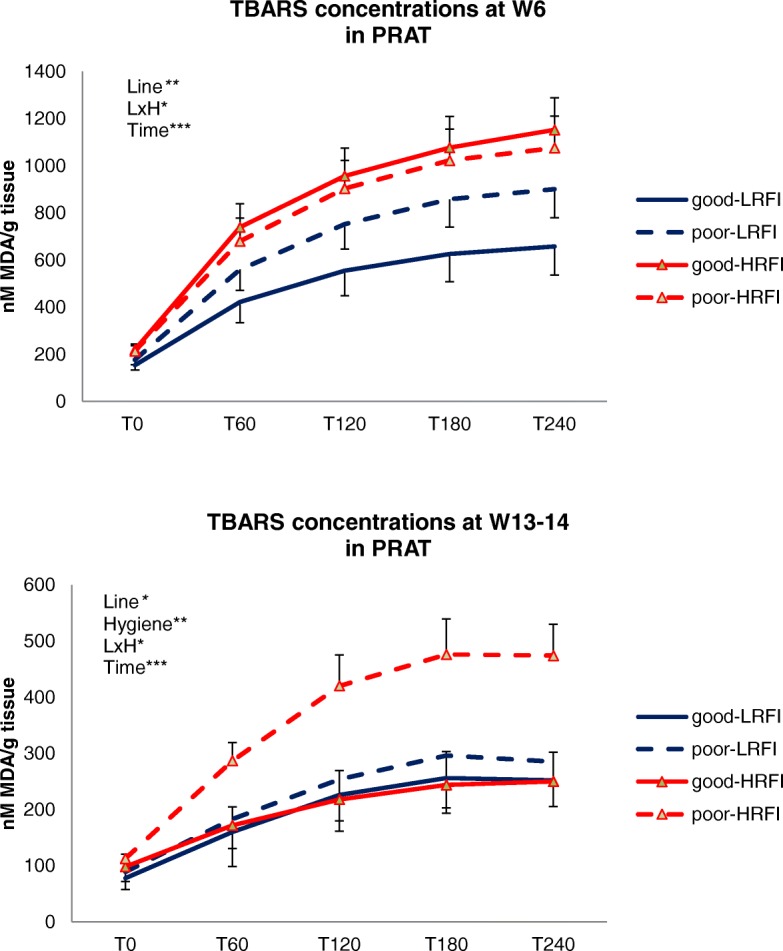
Effects of RFI line and hygiene conditions on lipid byproducts in adipose tissue. Pigs from two genetic lines divergently selected for low (LRFI) or high (HRFI) RFI were housed in good or poor hygiene conditions during 6 weeks (period 1, challenge). Half of these pigs were killed at week 6, whereas another half were placed in good hygiene conditions until slaughter at weeks 13 to 14 (period 2, recovery). Thiobarbituric reactive substances (TBARS) were measured in perirenal adipose tissue (PRAT) at different time points along forced chemical oxidation, and expressed as malondialdehydes (MDA) concentration (nM/g tissue). Effects of genetic line, hygiene conditions and their interaction (LxH) were tested for repeated measures data of TBARS along the kinetics. ***P < 0.001; ** *P* < 0.01; *P ≤ 0.05. Dashed lines are used for poor hygiene conditions, and continuous lines are used for good hygiene conditions; red color is used for high RFI pigs and blue colour is used for low RFI pigs

**Fig. 2:**
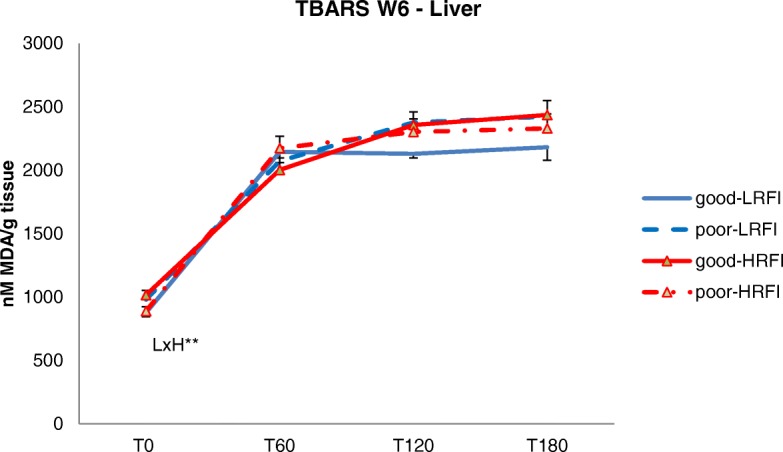
Effects of RFI line and hygiene conditions on lipid byproducts in the liver. Pigs selected for low (LRFI) or high (HRFI) residual feed intake (RFI) were housed either in good or poor hygiene conditions during 6 weeks. Thiobarbituric reactive substances (TBARS) in the liver were measured at different time points along a forced chemical oxidation kinetics, and expressed as malondialdehydes (MDA) concentrations (nM/g tissue). Effects of line, hygiene conditions and their interaction (LxH) were tested for repeated measures data of TBARS along the kinetics. LxH: interaction effect between genetic line (L) and hygiene conditions (H): **P = 0.01. Dashed lines are used for poor hygiene conditions, and continuous lines are used for good hygiene conditions; red color is used for high RFI pigs and blue color is used for low RFI pigs

At W6 (end of period 1), the HRFI pigs had fewer lipids in PRAT (*P* = 0.05) but more lipids in the LL muscle (*P* = 0.02) when compared with the LRFI pigs (Table 3). Poor hygiene conditions tended (*P* < 0.10) to reduce lipid content in PRAT, but did not alter total lipid contents in SCAT, liver and LL muscle as compared with good hygiene. The HRFI pigs exhibited greater thiobarbituric acid reactive substances (TBARS) levels (*P* < 0.01) in PRAT when compared with LRFI pigs (Fig. 1). There was also a difference in the response of line to hygiene degradation (Line x Hygiene, *P* = 0.02), so that poor hygiene conditions did not significantly alter TBARS concentrations in adipose tissue of HRFI pigs but increased their concentrations in LRFI pigs. No differences between the four groups were observed for TBARS concentrations in liver (Fig. 2), except at the beginning of the reaction (T0) when hepatic TBARS concentration was slightly increased by poor hygiene in the LRFI pigs but was decreased in the HRFI pigs (Line x Hygiene, *P* < 0.01).

**Table 3:** Effects of RFI line and hygiene of housing conditions on tissue lipid content in growing pigs

Line	Low RFI		High RFI			*P* values		
Hygiene	Good	Poor	Good	Poor	MSE	Line	Hygiene	LxH
Week 6 (*n* = 36)								
SCAT	61.7	59.2	60.0	59.1	7.2	0.71	0.48	0.73
PRAT	71.2	66.3	65.9	59.6	9.1	**0.05**	***0.08***	0.83
Liver	3.2	3.5	3.3	3.5	0.4	0.60	0.11	0.82
LL	0.81	0.70	0.95	1.06	0.30	**0.02**	0.99	0.30
Week 13–14 (*n* = 35)								
SCAT	77.2	70.7	69.4	67.5	5.8	0.12	0.45	0.96
PRAT	81.9	79.9	83.8	79.0	5.1	0.83	***0.07***	0.48
Liver	3.5	4.0	3.7	4.1	0.8	0.53	0.12	0.94
LL	0.88	0.99	1.36	1.23	0.35	**0.002**	0.33	0.52

Pigs from two genetic lines divergently selected for low (LRFI) or high (HRFI) RFI were housed either in good or poor hygiene conditions during the first 6 weeks after their transfer in growing-finishing pens (period 1). Half of these pigs were killed at week 6, whereas another half were placed in good hygiene conditions until slaughter at weeks 13 or 14 (period 2). Subcutaneous (SCAT) and perirenal (PRAT) adipose tissues, liver and *longissimus lumborum* (LL) muscle were sampled in pigs at the end points of the two periods. Lipid content was expressed in gram per 100 g of wet tissue. LxH: interaction between hygiene (H) and RFI line (L). MSE: root mean standard error of the statistical model. Bold face highlights significant differences (*P* ≤ 0.05) between treatments, and when italicized, this denotes a trend (0.05 < *P* ≤ 0.10)

At W13–14 (end of period 2), lipid content in PRAT was similar in LRFI and HRFI pigs, whereas intramuscular lipid content was again greater (*P* < 0.05) in HRFI pigs than in LRFI pigs (Table 3). At this stage, lipid content in PRAT still tended to be lower in pigs that were housed in poor conditions during the first period than in pigs always housed in good conditions. The TBARS levels measured in PRAT were higher in HRFI pigs than in LRFI pigs (Fig. 1). Importantly, the HRFI pigs reared in poor hygiene conditions during period 1 displayed greater TBARS levels in PRAT when compared with HRFI pigs never exposed to poor hygiene conditions, whereas there were no significant changes due to early hygiene conditions in the LRFI pigs (Line x Hygiene, *P* = 0.01).

### Antioxidant molecules in tissues

At W6 (end of period 1), the HRFI pigs displayed greater (*P* < 0.05) activity of the glutathione reductase (GSH-Rx) and catalase (CAT) in PRAT when compared with LRFI pigs (Table 4). In LL muscle, superoxide dismutase (SOD) activity also tended to be higher (*P* = 0.07) in HRFI pigs compared with LRFI pigs. In liver, antioxidant enzymes activities did not differ between the two lines. At this stage, poor hygiene conditions were associated with greater activities (*P* < 0.001 to *P* < 0.10) of the antioxidant enzymes (SOD, GSH-Rx and CAT) in PRAT when compared with good hygiene. Hygiene had almost no effects on antioxidant enzymatic defenses in lean tissues, except for SOD activity which was lower (*P* = 0.03) in liver of pigs housed in poor conditions. Oxidized (GSSG) and reduced (GSH) forms of glutathione had similar contents in the liver of all pigs (Table 4).

**Table 4:** Effects of RFI line and hygiene of housing conditions on antioxidant enzymes and glutathione content in tissues of growing pigs

Line	Low RFI		High RFI			*P* values		
Hygiene	Good	Poor	Good	Poor	MSE	Line	Hygiene	LxH
Week 6 (*n* = 36)								
SOD								
PRAT	81.4	109.4	99.5	104.8	26.1	0.44	***0.06***	0.17
Liver	2727	2289	2929	2520	551	0.25	**0.03**	0.94
LL	203	201	224	218	29.8	***0.07***	0.68	0.82
CAT								
PRAT	1002	1299	1169	1500	258	**0.04**	**0.001**	0.84
Liver (× 10^3^)	72.3	74.3	88.0	71.6	25.1	0.44	0.40	0.28
LL	1060	1116	1048	1020	193	0.41	0.83	0.52
GSH-Rx								
PRAT	77.9	107.1	110.5	133.4	20	**< 0.001**	**< 0.001**	0.64
Liver	5518	5214	5007	5098	631	0.16	0.63	0.37
LL	121	102	116	117	23.6	0.53	0.29	0.23
GSH - Liver	5389	6202	5318	5122	1433	0.68	0.91	0.34
GSSG - Liver	275	286	283	257	162	0.83	0.88	0.77
Ratio - Liver	23.7	29.2	26.4	21.9	14.8	0.66	0.93	0.35
Week 13–14 (*n* = 35)								
SOD								
PRAT	42.4	53.9	53.4	82.5	18.9	0.004	**0.004**	0.18
Liver	2594b	2392a	2507b	3257c	661	***0.09***	0.24	**0.05**
LL	221	218	241	240	30	**0.05**	0.85	0.89
CAT								
PRAT	781	810	812	1107	293	0.11	0.11	0.19
Liver (× 10^3^)	834	961	1004	923	180	0.29	0.72	0.10
LL	1006	960	852	945	200	0.23	0.73	0.31
GSH-Rx								
PRAT	52.2	67.9	59.8	89.3	23	***0.07***	**0.007**	0.38
Liver	4334	4783	5066	5391	672	**0.007**	0.11	0.78
LL	98	100	103	105	18	0.42	0.83	0.92

Pigs from two genetic lines divergently selected for low (LRFI) or high (HRFI) residual feed intake (RFI) were housed either in good or poor hygiene conditions during the first 6 weeks after their transfer in growing-finishing pens (period 1, challenge). Half of these pigs were killed at week 6, whereas another half were placed in good hygiene conditions until slaughter at weeks 13 or 14 (period 2, recovery). Activities of antioxidant enzymes such as glutathione reductase (GSH-Rx), superoxide dismutase (SOD) and catalase (CAT) were assessed (nM/min/g tissue) in perirenal adipose tissue (PRAT), liver and *longissimus lumborum* (LL) muscle. Reduced (GSH) and oxidized (GSSG) forms of glutathione (expressed as pmol per well) were also assessed in the liver. The ratio between reduced and oxidized forms (GSH:GSSG) was calculated. Letters (a,b,c) were added in case of interaction (LxH; *P* < 0.10) between hygiene (H) and RFI line (L), and means sharing a common letter did not differ. MSE: root mean standard error of the statistical model. Bold face highlights significant differences (*P* ≤ 0.05) between treatments, and when italicized, this denotes a trend (0.05 < *P* ≤ 0.10)

At W13–14 (end of period 2), the HRFI pigs had greater activities of SOD (*P* < 0.01) and GSH-Rx (*P* = 0.07) in PRAT when compared with the LRFI pigs (Table 4). The HRFI pigs also had greater GSH-Rx activity (*P* < 0.01) in liver, and there were tendencies for greater SOD activity (*P* < 0.10) in liver and LL muscle when compared with LRFI pigs. At this time, pigs housed in poor conditions during period 1 still had activated antioxidant enzymes activities in PRAT, namely SOD (*P* < 0.001) and GSH-Rx (*P* < 0.01), when compared with pigs never exposed to poor hygiene. Catalase activity in PRAT was similar in pigs whatever hygiene conditions of housing in period 1. In liver, early exposure to poor hygiene conditions was associated with greater SOD activity in HRFI pigs, but lower SOD activity in LRFI pigs at 13–14 weeks of age (Line x Hygiene, *P* = 0.05).

### Oxidative enzyme activities in adipose tissue and in vitro ROS production by adipocytes

At W6, the activities of mitochondrial enzymes involved in fatty acid oxidation (beta-hydroxyacyl-CoA dehydrogenase, HAD) and terminal nutrient oxidation (citrate synthase, CS) were similar in HRFI and LFRI pigs. Activity levels of these two enzymes were (*P* = 0.04) or tended (*P* = 0.07) to be higher in PRAT of pigs housed in poor hygiene conditions when compared with pigs housed in good conditions (Table 5). At W13–14, there was no difference in oxidative enzyme activities in PRAT between the four groups of pigs (Table 5).

**Table 5:** Effects of RFI line and hygiene conditions on oxidative enzymes in perirenal adipose tissue of growing pigs

	LRFI		HRFI				*P* values	
	Good	Poor	Good	Poor	MSE	Line	Hygiene	LxH
Week 6 (*n* = 36)								
HAD	4.42	5.55	4.42	5.60	1.59	0.96	**0.04**	0.96
CS	3.85	4.37	3.85	4.72	1.13	0.65	***0.07***	0.64
Week 13–14 (*n* = 35)								
HAD	3.78	4.12	3.56	4.53	1.4	0.84	0.17	0.51
CS	6.82	3.24	2.86	3.59	4.4	0.24	0.35	0.16

Pigs from two genetic lines divergently selected for low (LRFI) or high (HRFI) RFI were housed either in good or poor hygiene conditions during the first 6 weeks after their transfer in growing-finishing pens (period 1, challenge). Half of these pigs were killed at week 6, whereas another half were placed in good hygiene conditions until slaughter at weeks 13 to 14 (period 2, recovery). Enzymes related to oxidative nutrient catabolism, namely beta-hydroxy-acylCoA-dehydrogenase (HAD) and citrate synthase (CS) were measured (μmole/min/g tissue) in adipose tissue. LxH: interaction between hygiene (H) and RFI line (L). MSE: root mean standard error of the statistical model. Bold face highlights significant differences (*P* ≤ 0.05) between treatments, and when italicized, this denotes a trend (0.05 < *P* ≤ 0.10)

**Fig. 3:**
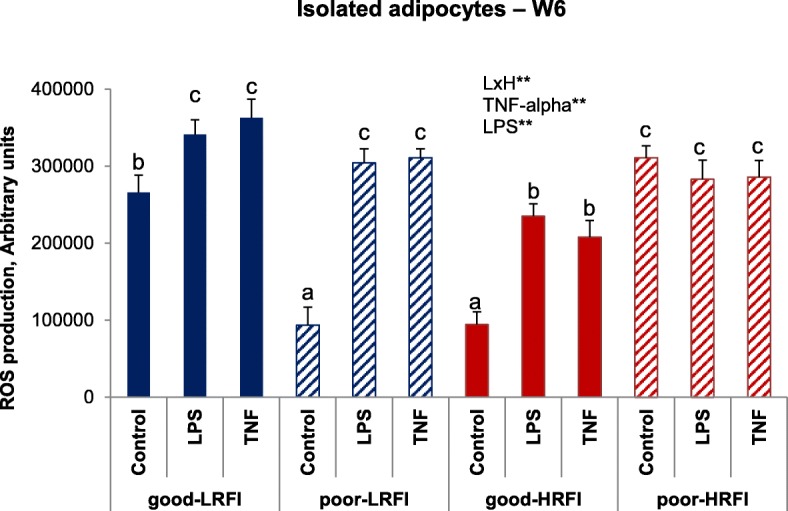
Reactive oxygen species (ROS) production in isolated mature adipocytes. Reactive oxygen species (ROS) production was determined in adipose cells isolated in pigs selected for low (LRFI) or high (HRFI) RFI and housed in good or poor hygiene conditions during 6 weeks. A fluorimetric assay using 2,7-dihydrochlorofluorescein (DHCF) was used to estimate ROS production into cells. Adipocytes were cultured in media with or without bacterial lipopolysaccharides (LPS: 1 mg/mL) or tumor necrosis factor (TNF-alpha: 1 ng/mL). A fluorimetric assay using 2,7-dihydrochlorofluorescein (DHCF) was used to estimate ROS production into cells. Data are expressed as mean ± S.E.M. (*n* = 8 pigs per group). LxH: interaction effect between genetic line (L) and hygiene conditions (H); LPS: effect of LPS; TNF-alpha: effect of TNF-alpha. **P < 0.01. Hatched charts are used for poor hygiene conditions, and full charts are used for good hygiene conditions; red color is used for high RFI pigs and blue color is used for low RFI pigs

At W6, production of reactive oxygen substrates (ROS) by mature isolated adipocytes was higher for the HRFI pigs facing poor hygiene conditions than when housed in good conditions (Fig. 3). On the opposite, ROS production by mature adipocytes was lower in LRFI pigs facing poor hygiene conditions than in LRFI pigs housed in good hygiene conditions (Line x Hygiene, *P* < 0.01). Addition of LPS or TNF-alpha to the media stimulated ROS production (P < 0.01), except in adipocytes harvested in poor-HRFI pigs where these reagents had no effects (Fig. 3).

## Discussion

### Short-term and long-lasting effects of poor hygiene on systemic and tissues redox states

Poor hygiene conditions lowered pig growth rate during the challenge period, and this reduced the development of visceral adiposity as indicated by the lower relative weight and lipid content of the perirenal fat. It has been established that poor environmental hygiene conditions induced a moderate inflammatory state, which was manifested by elevated plasma concentrations of haptoglobin [4, 5, 10], an acute phase protein whose concentration increases in inflammation. Therefore, the difference in adiposity observed in pigs housed in poor hygiene conditions might be due to an increased production of pro-inflammatory cytokines such as TNF-alpha, an adipose cytokine known to induce a fat mass loss during acute inflammatory diseases [11]. Finding greater dROM concentrations and lower total antioxidant capacity (FRAP) in plasma, the place where oxidative products from diverse origins accumulate, further indicated the occurrence of oxidative stress in pigs facing poor hygiene conditions, as defined as the redox imbalance due to excess of oxidants and depletion of antioxidants [12]. Similarly, an increase in plasma dROM and a decrease in total antioxidant capacity have been observed in piglets weaned in deteriorated conditions including the lack of cleaning and disinfection of rooms [13]. Oxidative stress (ROM values) and serum anti-oxidative capacity are also known to be associated with plasma concentrations of haptoglobin in young piglets exposed to inflammatory stimuli [14]. Another argument to support the occurrence of oxidative stress in response to hygiene degradation was the greater activities of antioxidant enzymes in perirenal adipose tissue of pigs housed in poor hygiene conditions as compared to pigs housed in good conditions. An up-regulation of antioxidant enzymes in adipose tissue was similarly observed in the early stages of human obesity, a disease associated with chronic and low grade inflammatory state [15], before the depletion of the sources of antioxidant enzymes in the later stages [16, 17]. In the present study, pigs facing poor hygiene conditions also exhibited greater activity levels of mitochondrial enzymes involved in oxidation (citrate synthase) and oxido-reduction process (beta-hydroxyl-acyl-CoA dehydrogenase) in perirenal adipose tissue, and for high RFI pigs, production of ROS by adipocytes was higher. Mitochondrial electron transfer for oxidative ATP regeneration is intrinsically linked to ROS production by the cells [18]. However, there is still a matter of debate if this mitochondrial metabolic activity can be the cause of the increased ROS production in adipocytes [19]. After a recovery period in clean conditions, pigs previously exposed to poor hygiene still tended to exhibit a lower lipid content in perirenal adipose tissue as compared with pigs never exposed to degraded hygiene, and the activation of antioxidant enzymes in perirenal adipose tissue was also maintained. This underlined the importance of early inflammatory events in the regulation of adipose tissue metabolism.

Importantly, perirenal fat was identified as the tissue that exhibited most of the changes due to hygiene conditions, at least when compared with subcutaneous adipose tissue, *longissimus* muscle and liver. Differences in growth dynamics, metabolism, and immune- and inflammation-related genes have been previously reported between perirenal and subcutaneous adipose tissues in pigs [20–22]. The responsiveness of antioxidant enzymes in adipose tissue has been also underlined in growing piglets exposed to a dietary methionine deficiency [23], an amino acid with important roles on redox metabolism. Differences between fat and lean tissues have been identified during the emergence of oxidative stress associated with obesity, so that antioxidant defenses were affected in adipose tissue but not in the liver [24]. Finally, numerous studies have pointed the role of adipose tissue physiology on systemic metabolism and immunity during physio-pathological diseases and deregulated metabolic states [19]. Adipocytes and adipose-resident immune cells affect each other in a metabolic-immune interface, so that metabolic features of adipose tissue and their regulations can provide both local and systemic effects, as shown in various diseases linked to low-grade inflammation [25]. Adipose tissue was also identified as an extrahepatic source of haptoglobin in humans with excess accumulation of body fat [26]; however, the situation might be different in young pigs [14]. Taken together, these data indicated that adipose tissue is an important site bridging energy, redox metabolism, immune and inflammatory stimuli. Nevertheless, it is noteworthy that at 13–14 weeks of age, there was a trend for a higher liver weight (relative to BW) in pigs having previously subjected to hygiene degradation during the first period of growth. Due to contribution of liver to detoxification, synthesis, and release of biomolecules, this may sign for modifications in protein metabolism due to hygiene degradation and deserves further studies.

### Genetic selection for feed efficiency altered redox metabolism in tissues and interacted with their responses to hygiene degradation

In the present study, two pig lines divergently selected for RFI, a measure of feed efficiency, were compared to test the hypothesis that genetic selection for production traits can affect redox metabolism in basal and (or) immune-stimulated conditions. Different authors have identified the response to oxidative stress or to oxygen level, inflammation, immune response and lipid metabolism as pathways differentially-regulated at the molecular levels between the two lines when compared in healthy conditions [9, 27]. Here, biochemical differences in redox metabolism between the two RFI lines were revealed in basal and challenged conditions. Especially, antioxidant enzymes activities were lower and TBARS concentrations were reduced in perirenal adipose tissue of the low RFI pigs (the most efficient) as compared with the high RFI pigs (the less efficient). Differences were still significant at weeks 13–14 in perirenal adipose tissue, and some differences between lines were also observed in the liver and muscle at this stage. This confirms the existence of tissue-specific differences in the ways different molecules participating to oxidative/antioxidant pathways were modulated by RFI divergence, as previously reported in different species [7–9]. The liver weight (relative to BW) was also lower in low RFI than in high RFI pigs at the two periods (challenge and resilience). This observation fits with the difference reported between these lines at different stages of growth [9, 28], and may be associated with difference in catabolic pathway activities between the two lines [28]. Importantly, the two lines responded somewhat differently to the hygienic challenge, so that not only growth rate was more affected in the high RFI pigs, but plasma dROM concentrations also increased specifically in these pigs when exposed to poor hygiene conditions. It has been reported that average daily gain, feed intake, feed efficiency and plasma haptoglobin concentrations were more affected by hygiene degradation in the high RFI pigs [5]. Adipocyte ROS production in response to pro-inflammatory situations were also different between the two RFI lines. First, adipocytes isolated from high RFI pigs exposed to poor hygiene produced more ROS than the other groups. Moreover, addition of TNF-alpha or liposaccharides (LPS) increased ROS production in all pigs, except in high RFI pigs housed in poor hygiene conditions. The increase in adipocyte ROS production due to pro-inflammatory reagents was in accordance to what is observed in murine 3 T3-L1 adipocytes [29, 30]. Because adipose cells from the high RFI pigs housed in poor hygiene conditions produced ROS at very high levels when cultured in the control medium, these cells might be desensitized to any re-stimulating inflammatory conditions such as TNF-alpha and LPS. After a recovery period, TBARS concentrations in perirenal adipose tissue were affected by early hygiene conditions in the high RFI pigs only, suggesting a longer lasting susceptibility to oxidative stress for the less efficient pigs. In agreement, long lasting effects of poor hygiene on lesions scoring (pleurisy, pericarditis) were greater in high RFI pigs than in low RFI pigs [5]. Altogether, these data support the view that pigs selected for low RFI may be less prone to oxidative stress as compared with high RFI pigs [8]. Because RFI is considered as a biological estimate of the net efficiency in nutrient utilization, this might be related to a better efficiency of mitochondria metabolism and changes in the mitoproteome [31] in low RFI pigs.

## Conclusions

Differences for redox metabolism in the perirenal adipose in both basal and challenged conditions were revealed between two porcine lines divergently selected for feed efficiency tissue. A lower susceptibility to oxidative stress was suggested for pigs selected for low residual feed intake, an observation that corroborates the lower inflammatory response and reduced growth disturbance previously reported in those pigs when facing poor hygiene conditions. This study demonstrates that selection for improved feed efficiency can modulate physiological systems important for adaptation and health. This could be of importance for pigs in production farms to limit economic losses and improve welfare. Among tissues, perirenal adipose tissue features highly responded to hygiene degradation, suggesting that this tissue could play an important role in the management of oxidative stress when pigs are facing pro-inflammatory environmental conditions.

## Methods

### Ethics

Animals were cared in compliance with the French directive on animal experimentation, and they were killed in compliance with national regulations and according to procedures approved by the French Veterinary Services at INRA experimental facilities. The study was submitted to and approved by our institutional ethics committee (Comité Rennais d’Ethique en matière d’Expérimentation Animale, CREEA, Rennes, France) and authorized by the French Ministry of National Education, Higher Education and Research.

### Animals

The trial included 71 Large-White pigs produced from the 8th generation of a divergent genetic selection for RFI [32]. The RFI represents the difference between observed and expected feed intake of an animal based on the estimated requirements for its maintenance and growth. The RFI criterion was calculated as follows:

$$ \mathrm{RFI}=\mathrm{ADFI}\hbox{-} \left(1.24\times \mathrm{ADG}\right)\hbox{-} \left(31.9\times \mathrm{BFT}\right), $$

where ADFI is the average daily feed intake (g/day) between 35 and 95 kg BW, ADG is the average daily gain (g/day) during the same period, and BFT is the backfat thickness (mm) used as a surrogate of adiposity at 95 kg BW. The objectives of the selection were keeping ADG and BFT constant between the two lines [32]. For selection, the RFI of boars was computed using their own records for ADFI, ADG and BFT, and taking into account the fixed effects of contemporary group and pen size. The R^2^ of the model to compute the predicted feed intake of P1 animals was 0.66 [33]. As compared with pigs selected for high RFI (HRFI), pigs selected for low RFI (LRFI) have an improved gain-to-feed ratio, which is a widely used indicator of feed efficiency.

All pigs were born in the INRA experimental facilities (Unité Experimentale Porcs de Rennes, 35,590 Saint-Gilles, France) and weaned at 4 weeks. The disease status of the RFI herd was negative for *Actinobacillus* and porcine reproductive and respiratory syndrome virus (PRRS), but it was positive to porcine respiratory Coronavirus and *Mycoplasma hyopneumonia*e. The experiment began when pigs reached 12 weeks of age, hereafter referred as Week 0 [W0]. The experiment was divided in two periods: a challenge period (Period 1) and a recovery period (Period 2). The challenge period (Period 1) started on W0 and lasted 6 weeks. Pairs of littermates (same sex: male or female) were constituted within each RFI line (*n* = 40 for low RFI and *n* = 32 for high RFI pigs). Within each pair, animals were assigned to one of the two groups: good or poor hygiene conditions. One animal died from acute diarrhoea soon after the beginning of the experiment. Therefore, a total of 36 males and 35 females was finally considered: 8 males and 7 females for HRFI pigs housed in good conditions, 8 males and 8 females for HRFI pigs housed on poor conditions, 10 males and 10 females for LRFI housed in good conditions, and 10 males and 10 females for LRFI pigs housed in poor conditions. Housing procedures were derived from Le Floc’h and colleagues [4] and details on how poor or good hygiene conditions were established are available in our associate publication [5]. Briefly, the poor conditions consisted of no cleaning nor sanitation of the room after previous occupation by non-experimental pigs, a decreased aeration rate and no biosecurity precaution; experimental pigs in poor housing conditions were also mixed with non-experimental pigs during the challenge period. Conversely, the aeration rate and temperature were optimal in clean conditions, and the room was disinfected; strict biosecurity precautions were applied, including no mixing of the experimental pigs with non-experimental pigs. At W6, half of the pigs (pairs of pigs balanced for the sex, line and hygiene conditions) was killed. The recovery period (Period 2) lasted from W6 to W13–14. During this period, all the remaining pigs were housed in good conditions until slaughter.

Throughout the experimental period, pigs were housed in individual pens (85 × 265 cm) on concrete floor. They were fed ad libitum a standard growing diet used for selection (94.7 kJ digestible energy; tryptophane/lysine 0.2; lysine 8.3 g/kg; dry matter 87.38%; starch 44.17%; neutral detergent fibre 15.7%; acid detergent fibre 5.6%; acid detergent lignin 1.6%; fat 3.14%; wheat 32.2%; barley 30%; maize 15%; soya bean 7%; bran 5%). They had free access to water. Pigs were weighed after an overnight fast at W0, W6, W12, and just before slaughter at W13 or W14. Feed consumption was estimated weekly as the difference between allocated feed minus feed refusals.

### Blood and tissues collection

The day before slaughter at W6 or W13–14, and after an overnight fast, blood was collected between 08:00 h and 10:00 h by jugular punctures in heparin (9 ml) or ethylene-diamine-tetra-acetic acid (EDTA) vacutainers tubes (4 and 9 ml). Samples were kept on ice, until centrifugation (2500 x *g*, 4 °C, 15 min), and plasma was aliquoted and kept at − 80 °C until analyses. In the next morning, the pigs were weighed. Then, they were transported to the experimental slaughterhouse (Unité Experimentale Porcs de Rennes, INRA, Saint Gilles, France), to be euthanatized by electrical stunning and exsanguination. Animals of each group were alternatively killed. The liver and the entire PRAT were removed and weighed. A sample (50 to 100 g) of the LL muscle was also collected at the level of the last rib from the right side of the carcass within 30 min after pig death. Tissue samples were cut into small pieces (approximately 0.5 × 0.5 × 0.5 cm), snap frozen in liquid nitrogen, and stored at − 76 °C until metabolic analyses. Portions (100 g) of liver, PRAT and LL muscle were also kept at − 20 °C in vacuum plastic bags; liver and LL were subsequently freeze-dried and stored again at − 20 °C. For pigs at W6, about 15 g of adipose tissue was also put in warm Krebs-Ringer buffer before digestion for cellular isolation. The day after slaughter, dorsal SCAT was collected from the left side of the carcass and weighed; a portion (100 g) was kept at − 20 °C in a vacuum plastic bag. The LL muscle was also dissected from the loin of the same carcass side, and weighed. Organ and tissue weights were expressed relative to BW.

### Total lipid content and TBARS index

Sub-samples of frozen (PRAT and SCAT) and freeze-dried (LL and liver) tissues were ground in a cutter mill (Grindomix GM200, Retsch, Newton, PA). In PRAT and SCAT (500 mg each) and in LL muscle (approximately 800 mg), total lipid content was determined by the application of supercritical CO_2_ and solvent extraction [34] with an automatic system (Leco TFE 2000 Instrument, Leco, St. Joseph, MI). In liver (approximately 2.5 g of sample), lipid content was determined using a chloroform and methanol (2:1) extraction procedure [35]. Results were expressed in gram of lipids per 100 g of wet tissue weight.

The TBARS concentration was determined in PRAT and liver samples submitted to different incubation times (0, 60, 120, 180 and 240 min) at 37 °C, with a forced chemical oxidation induced by iron trichloride and sodium ascorbate [36]. The TBARS concentration was expressed in nmoles of malondialdehyde (MDA) produced per g of tissue.

### Antioxidant enzyme activities

The activities of CAT, total SOD and GSH-Rx were determined in liver, PRAT and LL (approximately 100 mg each) after tissue homogenization in 0.8 mL ice-cold sucrose buffer (0.5 M) containing 0.05 M Tris-HCl and 1 mM EDTA (pH 7.4). The CAT activity was measured by spectrometry at 240 nm using an Uvikon Bio-Tek apparatus (Secomam, Alès, France) following the decrease in H_2_O_2_ concentration at 25 °C [37]. Total SOD activity was measured at 450 nm by the inhibition of the xanthine/xanthine oxidase-mediated oxidation of cytochrome-c, using a dedicated kit (19,160 SOD; Sigma Aldrich, St. Louis, MO, USA) with a SOD standard from bovine erythrocytes (S7571-15KU; Sigma Aldrich) and a microplate reader (Thermo-Labsystems, Franklin, MA). Activity of GSH-Rx was assessed at 37 °C by following NADPH decrease at 340 nm absorbance [38], using a KoneLab 20i analyzer (Thermo Scientific, Cergy Pontoise, France). All enzyme activities were expressed as units/min and per g of tissue wet weight.

### Oxidative enzyme activities

Activities of HAD and CS were measured in PRAT. Briefly, adipose samples (300 mg each) were homogenized in phosphate buffer (pH 7.4) containing 2 mM EDTA. Mixtures were sonicated (60 s, 50 Hz; Bioblock scientific, Illkirch, France), centrifuged (13 min at 1500 g at 4 °C; Mikro 200R Hettich, Sigma-Aldrich, St. Louis, MO, USA), and supernatants were stored on ice. Activities were immediately assayed by spectrometry in the KoneLab analyzer at 30 °C following dedicated methods, at 340 nm absorbance for HAD [39] and at 405 nm for CS [40], respectively, and expressed as μmole/min/g tissue.

### Glutathione content

Contents in GSH and GSSG were enzymatically analyzed in liver [41]. Briefly, samples (100 mg) were homogenized in ice-cold buffer with 2 mL 5-sulfosalicylic acid (5% w/v) and centrifuged for 5 min at 10000 g at 4 °C. The supernatants were collected and analyzed using the Glutathione Assay Kit (7511–100-K; Trevigen, Gaithersburg, MD, USA) and the microplate reader. Contents are expressed as pmol of glutathione per well.

### Plasma redox

The dROM contents were measured in plasma samples (250 μL) on the Konelab analyzer by the dROM test (Diacron, Grosseto, Italy), described as a simple assay for analyzing the total amount of hydrogen peroxides [42]. Total antioxidant activity in plasma was estimated by FRAP method [43], which was based on the reduction of the 2,4,6-tripyridyl-*s*-triazine (TPTZ) with ferric chloride hexahydrate producing blue ferrous complexes producing intense blue color ferrous complexes. The final absorbance was measured at 595 nm after 30 min incubation at 37 °C in the darkness [44], using a microplate reader (Thermo-labsystems, Franklin, MA). Measurements were performed in triplicates and results were expressed as molar Trolox equivalents antioxidant capacity (TEAC) per L.

### ROS production by adipocytes

From a subset of pigs slaughtered at W6 (*n* = 8 per group), mature adipocytes were isolated following standard procedures [45], by digestion of adipose tissue samples (15 g) in HEPES buffer (2 mL/g of tissue) containing 2% BSA and 2 mg/mL collagenase A. A fivefold excess of buffer free of collagenase was added, before cells were centrifuged at 400×g for 10 min. Floating adipocytes were immediately placed into culture media with or without TNF-alpha (1 ng/mL; Invitrogen-Life Technologies, Saint-Aubin, France) or Gram-negative bacterial lipopolysaccharides (LPS; 1 mg/mL; *Escherichia coli* O55:B5, Sigma-Aldrich, St-Louis, MO) to induce an inflammatory response. The production of ROS by the cells in the different culture conditions was determined by a fluorimetric assay after 90 min of incubation with 5 μM of DHCF [46]. The fluorescence was measured with a multi-detection microplate reader (Mithras, LB 940, Berthold Technologies, Bad Wildbad, Germany) using an excitation and emission light at 485 nm and 535 nm, respectively. Samples were also incubated in the presence of 50 U of PEG-SOD, which was used as negative control to assess the specificity of the assay.

### Statistical analyses

Statistical analyses were carried out separately for each period (period 1: challenge; period 2: resilience) using the SAS software (SAS Institute, Cary NC, New-York), and considering pig as the experimental unit. For all traits except adipocyte ROS production and TBARS concentrations, GLM procedures were used to analyze data with models including hygiene conditions (good or poor), line (LRFI or HRFI), and the interaction between hygiene conditions and line as the main effects. How initial BW may also influence final BW and ADG was tested using initial BW as a covariable in the models. For ROS production, the model included hygiene conditions (poor or good), line (LRFI or HRFI), and presence of LPS (yes or no) or TNF-alpha (yes or no), and all the interactions between the main factors. For TBARS concentrations, a MIXED model was used with hygiene conditions, line and the interaction between hygiene conditions and line as the main effects, and a repeated statement [47] to indicate that data are correlated on the same animal during the forced oxidation kinetics. Interaction effects between line or hygiene conditions and time were also tested but were not significant. *P* ≤ 0.05 was considered significant, and 0.05 < *P* ≤ 0.10 was discussed as a trend.

## Supplementary information

**Additional file 1: Table S1.** Effects of RFI line and hygiene conditions on tissue weights of pigs. Weights of adipose tissues collected at the perirenal (PRAT) or subcutaneous (SCAT) locations, loin skeletal muscle (LL: *longissimus lumborum*) and liver in pigs at the two time points (week 6 and week 13–14) are given in this additional Table.

## Data Availability

Individual data used/analysed in this study are available from the corresponding author on reasonable request.

## Abbreviations

ADG: Average daily gain
BFT: Backfat thickness
BW: Body weight
CS: Citrate synthase
dROM: Diacron reactive oxygen metabolites
EDTA: Ethylene-diamine-tetra-acetic acid
FRAP: Ferric reducing ability of plasma
GGSG: Oxidized form of glutathione
GSH: Reduced form of glutathione
GSH-Rx: Glutathione reductase
HAD: Beta-hydroxy-acyl-CoA-dehydrogenase
HRFI: High residual feed intake
LL: *Longissimus lumborum* muscle
LPS: Bacterial liposaccharides
LRFI: Low residual feed intake
MDA: Malondialdehydes
MSE: Mean standard error
NADPH: Nicotinamide adenine dinucleotide phosphate, reduced form
PRAT: Perirenal adipose tissue
RFI: Residual feed intake
ROS: Reactive oxygen species
SCAT: Subcutaneous backfat adipose tissue
SOD: Superoxide dismutase
TBARS: Thiobarbituric acid reactive substances
TNF-alpha: Tumor necrosis factor-alpha
W: Week
