# Supplementary material for: Tissue-specific responses of antioxidant pathways to poor hygiene conditions in growing pigs divergently selected for feed efficiency
Source: BMC Vet Res. 2019 Oct 16;15:341. doi: 10.1186/s12917-019-2107-2 (PMC6794813; doi:10.1186/s12917-019-2107-2)
Supplement: Supplementary file 1 — Additional file 1: Table S1. Effects of RFI line and hygiene conditions on tissue weights of pigs. Weights of adipose tissues collected at the perirenal (PRAT) or subcutaneous (SCAT) locations, loin skeletal muscle (LL: longissimus lumborum) and liver in pigs at the two time points (week 6 and week 13–14) are given in this additional Table. [file 12917_2019_2107_MOESM1_ESM.docx]

**Additional Table 1 Effects of RFI line and hygiene conditions on tissue weights of pigs**

| **Line** | **Low RFI** | | **High RFI** | |  | ***P* values** | | |
| --- | --- | --- | --- | --- | --- | --- | --- | --- |
| **Hygiene** | **Good** | **Poor** | **Good** | **Poor** | **MSE** | **Line** | **Hygiene** | **LxH** |
| **Week 6 (n = 36)** | | | | | | | | |
| SCAT | 1116 | 1001 | 942 | 805 | 248 | **0.03** | 0.14 | 0.89 |
| PRAT | 338 | 281 | 253 | 197 | 74 | **0.002** | **0.03** | 0.97 |
| Liver | 1378 | 1363 | 1383 | 1331 | 189 | 0.83 | 0.60 | 0.77 |
| LL | 1390 | 1399 | 1148 | 1023 | 176 | **<0.001** | 0.33 | 0.26 |
| **Week 13-14 (n = 35)** | | | | | | | | |
| SCAT | 2793 | 2358 | 2712 | 1884 | 653 | 0.23 | **0.009** | 0.39 |
| PRAT | 954b | 881b | 1000b | 611a | 244 | 0.19 | **0.009** | ***0.07*** |
| Liver | 1790 | 1727 | 2180 | 1901 | 236 | **0.001** | **0.04** | 0.19 |
| LL | 2635 | 2393 | 2514 | 1916 | 331 | **0.01** | **0.001** | 0.13 |

Pigs from two lines divergently selected for low (LRFI) or high (HRFI) RFI were housed either in good or poor hygiene conditions during the first 6 weeks (W) after their transfer in growing-finishing pens (period 1, challenge). Half of these pigs were killed at week 6 (W6: n = 20 LRFI pigs in good conditions, n = 20 HRFI pigs in good conditions, n = 15 LRFI pigs in poor conditions, n = 16 HRFI pigs in poor conditions). The other halves were placed in good hygiene conditions until slaughter at weeks 13 to 14 (period 2, recovery: n = 10 LRFI pigs from good conditions, n = 10 HRFI pigs from good conditions, n = 7 LRFI pigs from poor conditions, n = 8 HRFI pigs from poor conditions). Tissues (LL: *longissimus lumborum*; PRAT: perirenal adipose tissue, SCAT: subcutaneous adipose tissue) were weighed (g) at slaughter. Letters (a,b,c) were added in case of interaction (LxH; *P* < 0.10) between hygiene (H) and RFI line (L), and means sharing a common letter did not differ. MSE: root mean standard error of the statistical model. Bold face highlights significant differences (*P* ≤ 0.05) between treatments, and when italicized, this denotes a trend (0.05 < *P* ≤ 0.10).
